# Factors influencing receipt of specialty neurologic care for patients with Huntington's disease in Colorado

**DOI:** 10.1016/j.prdoa.2026.100473

**Published:** 2026-06-19

**Authors:** Amy C. Ogilvie, Emily Forbes, Chun Chieh Lin, James F. Burke, Hillary D. Lum, Sean M. Reed

**Affiliations:** a Health Services Research Division, Department of Neurology, The Ohio State University Wexner Medical Center, 395 W 12^th^ Ave, Columbus, OH 43210, USA; b Division of General Internal Medicine, Department of Medicine, University of Colorado Anschutz Medical Campus, Academic Office One, 12631 East 17^th^ Ave, Aurora, CO 80045, USA; c Department of Neurology, University of Colorado Anschutz Medical Campus, Research Complex 2, 12700 East 19^th^ Ave, Aurora, CO 80045, USA; d Division of Geriatric Medicine, Department of Medicine, University of Colorado Anschutz Medical Campus, Academic Office One, 12631 East 17^th^ Ave, Aurora, CO 80045, USA; e College of Nursing, University of Colorado Anschutz Medical Campus, Education II North, 13120 East 19^th^ Ave, Aurora, CO 80045, USA

**Keywords:** Huntington disease, Patient care, Neurologists, Health services research

## Abstract

**Background:**

Despite recognized importance of specialized neurological care for patients with Huntington's disease (HD), little is known about the number or characteristics of patients who receive this type of care.

**Objectives:**

To identify differences between patients receiving care from a movement disorders specialist (MDS) compared to a general neurologist using data from the Colorado All Payers Claims Database between 2019 and 2022.

**Methods:**

Logistic regression models were used to compare characteristics of patients who had received care from a MDS compared to a general neurologist in a given year.

**Results:**

Approximately half (49.9%) of patients with HD received care from any neurologist and a third (33.8%) received care from a MDS. There were no differences in characteristics between patients who received MDS care compared to general neurologist care.

**Conclusions:**

Practice-related characteristics and patient perspectives on care access warrant further study to clarify barriers to specialized neurological care.

## Highlights

• The Colorado All Payers Claims Database offers a unique perspective to assess healthcare utilization across patients with Huntington's disease across multiple insurance payers.
• Approximately half (49.9%) of patients with Huntington's disease received care from any neurologist during a given calendar year.
• Approximately one third (33.8%) of patients with Huntington's disease received care from a movement disorder specialist during a given calendar year.

## 1 Introduction

Huntington's disease (HD) is a neurodegenerative disease that causes progressive motor, cognitive, and psychiatric symptoms over 15–20 years [1]. Since there are no disease-modifying treatments or cures, patients rely on symptom management to reduce disease burden and minimize impacts on quality of life. Expert consensus has described a specialized approach to care for patients with HD as the gold standard of care, with a neurologist often serving as the primary managing physician [2]. Movement disorders clinics often have specific capacity to provide patients with specialized care, with neurologists trained in the assessment and management of common HD symptoms [3]. Studies describe benefits of receiving care from a movement disorder specialist compared to a general neurologist, such as a decreased risk of admission to long-term care [4].

While specialized care benefits patients with HD, geographic barriers limit access for many. Most neurologists are clustered in urban areas or tertiary care centers, disadvantaging rural patients [5]. As HD progresses, patients may have greater difficulty in traveling to specialty clinics due to decreased functional capacity. Notably, over 40% of patients with HD over the age of 65 live at least 100 miles from the nearest HD Center of Excellence, indicating the potential travel burden patients with later stages of disease may experience [6].

This study increases understanding of the use of specialized neurological care among patients with HD by identifying clinical and sociodemographic differences in the receipt of care from a movement disorders specialist (MDS) compared to a general neurologist using data from the Colorado All Payers Claims Database (APCD). The Colorado APCD provides comprehensive statewide data for a broader population of patients living with HD than health records from any one clinic or healthcare system. By establishing baseline evidence on patterns of neurological care for patients with HD in Colorado, this study provides the baseline evidence needed to support national-level research examining specialized movement disorders care utilization and the impact on patient outcomes in HD.

## 2 Methods

### 2.1 Study population

This cross-sectional study used data from a Colorado APCD de-identified data set for 2019–2022 and was determined to be non-human subjects research by the Colorado Multiple Institutional Review Board. The Colorado APCD is a collection of medical (including durable medical equipment), dental, and pharmacy insurance claims information from patients seeking care with multiple insurance plans, including commercial insurance, Medicare, and Colorado's Medicaid program [7]. Individuals not included within the APCD are those on federal insurance programs (e.g., Veterans Affairs or Indian Health Services), the majority of ERISA-based self-insured employers, and uninsured or self-pay individuals. In 2023, the dataset represented 70% of covered lives in Colorado.^7^ Patients with HD were identified based on the presence of at least one medical or pharmaceutical claim with an International Classification of Diseases, 10th Edition (ICD-10) code for HD, “G10” [8]. Patients were 21 years of age or older at the end of the calendar year to include only patients with likely adult-onset HD. If a patient with HD had multiple years of data, one year of data was chosen at random for inclusion in these analyses.

### 2.2 Variables of interest

To assess access to specialty neurological care, the primary outcome of this study was level of specialized neurological care received over a given calendar year. Levels of neurological care included MDS, general neurologist but not MDS, only PCP, and no neurology nor PCP care and were defined as at least one medical claim with the given provider. Patients were grouped based on the highest level of specialized neurological care received during the calendar year. First, it was determined whether a patient had received care from an MDS. MDS care was defined as having at least one claim associated with a board-certified neurologist with fellowship training in movement disorders, determined by cross-verifying the neurologist first and last name with the Parkinson's Foundation list of MDSs [9]. For neurologists not on the list, we cross-verified information using their institution's website. Board certification as a neurologist was determined based on the presence of a National Uniform Claim Committee taxonomy code of 2084N0400X [10]. If a patient did not receive care from an MDS, it was then determined whether they had care provided by a general neurologist. Care by a general neurologist was defined by having at least one claim during the calendar year associated with a board-certified neurologist who did not have additional MDS training. If a patient had not received care from any type of neurologist, it was then determined whether they had received care from a PCP. Care from a PCP was defined as having at least one claim associated with a provider who had a taxonomy code of 207Q00000X, 363LF0000X, or 363LP2300X (specialization in family medicine or primary care).

The primary exposure was rurality of patient residence, determined using the Colorado Rural Health Center's 2022 County Designations (Urban, Rural, Frontier) [11]. Due to limited sample size in Frontier areas, we combined Rural and Frontier into one rural category. Additional covariates included patient age, sex, race/ethnicity, disease stage, Charlson Comorbidity Index (CCI) score, and insurance coverage. Disease staging criteria were based on a previously developed and well-cited algorithm for determining disease stage among patients with HD utilizing administrative claims data and expert MDS consultation [8]. Late-stage disease was determined by at least one of: skilled nursing facility care, feeding tube placement, pressure ulcers, aspiration pneumonia, respiratory failure, cachexia, at least two falls in a one-month period, dysarthria, contractures, use of a wheelchair, hospital bed, or lift, or use of hospice care. Middle-stage disease required absence of late-stage disease indicators plus at least one of: dementia, gait disturbance, any fall during the calendar year, dysphagia, use of physical therapy, use of speech therapy, home assistance, or walker use. Remaining patients were classified as early-stage.

### 2.3 Data analysis

Initially we compared neurologic care levels using Chi-square tests for categorical variables and ANOVA for continuous variables. Logistic regression models were used to make three different comparisons: (1) MDS vs general neurologist, (2) any neurologist vs only PCP, and (3) any neurologist vs no neurologist nor PCP. Unadjusted models were performed for each of the covariates listed above followed by a fully adjusted model. We set statistical significance at *p* < 0.05 and performed all analyses using R version 4.5.1.

## 3 Results

We identified 527 patients with HD who received care in Colorado between 2019 and 2022. Approximately half (49.9%) received neurologist care, with 33.8% seeing a MDS during the given calendar year. Table 1 describes the patient population. Neurological care differed significantly by age, CCI, insurance coverage, and disease stage.

**Table 1:** Characteristics of patients with HD by highest level of neurological care received.

	Overall (*N* = 527)	MDS (*N* = 178)	General Neurologist (*N* = 85)	Only PCP (*N* = 166)	No Neurologist nor PCP (*N* = 98)	*P*-Value
Mean (SD)						
Age	58.5 (15.8)	55.0 (15.6)	61.5 (13.0)	60.9 (15.4)	58.5 (17.9)	**0.001**
CCI	2.8 (3.0)	2.3 (2.8)	3.5 (3.3)	3.3 (3.1)	2.6 (2.4)	**0.001**
N (%)						
Sex – Female	266 (50.5)	94 (52.8)	43 (50.6)	80 (48.2)	49 (50.0)	0.863
Race/Ethnicity – Non-White NH	171 (32.4)	62 (34.8)	24 (28.2)	49 (29.5)	36 (36.7)	0.454
Insurance						**0.005**
Commercial	183 (34.7)	71 (39.9)	24 (28.2)	57 (34.3)	31 (31.6)	
Medicaid	136 (25.8)	44 (24.7)	22 (25.9)	33 (19.9)	39 (39.8)	
Medicare	208 (39.5)	63 (35.4)	39 (45.9)	76 (45.8)	28 (28.6)	
Rural Residence	76 (14.4)	20 (11.2)	12 (14.1)	33 (19.9)	11 (11.2)	0.098
Disease Stage						**< 0.001**
Late-Stage	212 (40.2)	50 (28.1)	36 (42.4)	95 (57.2)	31 (49.0)	
Mid-Stage	137 (26.0)	69 (38.8)	29 (34.1)	20 (12.0)	19 (19.4)	
Early Stage	178 (33.8)	59 (33.1)	20 (23.5)	51 (30.7)	48 (49.0)	
Year						0.156
2019	156 (29.6)	49 (27.5)	26 (30.6)	42 (25.3)	39 (39.8)	
2020	126 (23.9)	41 (23.0)	16 (18.8)	49 (29.5)	20 (20.4)	
2021	122 (23.1)	40 (22.5)	23 (27.1)	35 (21.1)	24 (24.5)	
2022	123 (23.3)	48 (27.0)	20 (23.5)	40 (24.1)	15 (15.3)	

HD – Huntington's Disease; MDS – Movement Disorder Specialist; PCP – Primary Care Provider; SD – Standard Deviation; CCI – Charlson Comorbidity Index; NH – Non-Hispanic.

There were no significant differences in the adjusted model comparing MDS care to general neurologist care (Table 2). Rural residence was associated with lower odds of receiving any neurologist care compared to only PCP care (OR (95% CI): 0.46 (0.26, 0.82)). In the same adjusted model, middle-stage patients had greater odds of receiving any neurologist care compared to early-stage patients (OR (95% CI): 3.79 (2.04, 7.24)). A similar pattern was observed in the adjusted model comparing any neurologist care to no neurologist nor PCP care (Table 2), with middle-stage patients having greater odds of receiving neurologist care (OR (95% CI): 3.91 (2.07, 7.63)). In this model, late-stage patients had greater odds of receiving neurologist care (OR (95% CI): 2.07 (1.11, 3.90)) while Medicaid insurance (OR (95%CI): 0.39 (0.21, 0.73)) and increasing age (OR (95% CI): 0.97 (0.94, 0.99)) were associated with lower odds of receiving any neurological care.

**Table 2:** Logistic regression models for comparisons regarding highest level of specialized neurological care.

	MDS vs General Neurologist		Any Neurologist vs Only PCP		Any Neurologist vs No Neurologist nor PCP	
	Unadjusted OR (95% CI)	Adjusted OR (95% CI)	Unadjusted OR (95% CI)	Adjusted OR (95% CI)	Unadjusted OR (95% CI)	Adjusted OR (95% CI)
Rural Residence	0.77 (0.36, 1.70)	0.76 (0.34, 1.76)	**0.56** **(0.33, 0.95)**	**0.46** **(0.26, 0.82)**	1.10 (0.54, 2.36)	1.08 (0.50, 2.46)
Age	**0.97** **(0.95, 0.99)**	0.98 (0.95, 1.00)	**0.98** **(0.97, 1.00)**	0.98 (0.96, 1.00)	0.99 (0.98, 1.01)	**0.97** **(0.94, 0.99)**
Female Sex	1.09 (0.65, 1.84)	1.09 (0.64, 1.87)	1.17 (0.79, 1.73)	1.14 (0.75, 1.73)	1.09 (0.68, 1.73)	1.14 (0.70, 1.86)
Non-White NH Race/Ethnicity	1.36 (0.78, 2.42)	1.15 (0.63, 2.11)	1.16 (0.76, 1.78)	1.07 (0.68, 1.70)	0.84 (0.52, 1.37)	0.88 (0.52, 1.48)
Commercial Insurance	Reference	Reference	Reference	Reference	Reference	Reference
Medicaid	0.69 (0.34, 1.40)	0.52 (0.24, 1.13)	1.16 (0.69, 1.99)	1.14 (0.62, 2.10)	**0.54** **(0.30, 0.94)**	**0.39** **(0.21, 0.73)**
Medicare	**0.54** **(0.29, 0.99)**	0.68 (0.35, 1.29)	0.82 (0.53, 1.28)	1.13 (0.69, 1.88)	1.21 (0.68, 2.18)	1.26 (0.68, 2.35)
Early Stage	Reference	Reference	Reference	Reference	Reference	Reference
Mid Stage	0.81 (0.41, 1.57)	1.15 (0.56, 2.37)	**3.16** **(1.77, 5.84)**	**3.79** **(2.04, 7.24)**	**3.13** **(1.73, 5.87)**	**3.91** **(2.07, 7.63)**
Late Stage	**0.47** **(0.24, 0.91)**	0.74 (0.35, 1.54)	**0.58** **(0.37, 0.92)**	0.66 (0.39, 1.12)	1.69 (0.98, 2.93)	**2.07** **(1.11, 3.90)**
CCI	**0.88** **(0.80, 0.96)**	0.96 (0.84, 1.09)	**0.93** **(0.87, 0.99)**	1.02 (0.92, 1.12)	1.01 (0.93, 1.09)	1.06 (0.93, 1.22)

MDS – Movement Disorder Specialist; PCP – Primary Care Provider; NH – Non-Hispanic; CCI – Charlson Comorbidity Index.

Bolded values indicated statistical significance at a p-value less than 0.05.

Reference indicates the value of a categorical variable used as the reference group within the logistic regression model.

## 4 Discussion

In this state-level analysis, approximately half (49.9%) of patients with HD received neurologist care, lower than rates reported for Parkinson's disease (PD), though patients with HD more often saw a MDS [12]. HD's rarity and the availability of predictive genetic testing may drive patients toward specialized care. However, only a third of patients with HD received care from a MDS (33.8%). We identified no differences in characteristics between patients who received care from a MDS compared to a general neurologist. Additional exploration of practice-related characteristics, such as specialist wait times, and patient perspectives on their decisions to access neurological care is needed to better understand potential barriers and facilitators to receipt of neurological care.

Patients living in rural areas had lower odds of receiving any neurologist care compared to only PCP care, indicating that patients in these areas may more heavily rely on their PCPs to help manage their symptoms. As neurologists tend to cluster in urban areas, PCPs are often more accessible in rural areas and may be particularly beneficial for more urgent needs. Middle-stage patients with HD had the highest odds of neurologist utilization, with 71.5% of middle-stage patients receiving care from a neurologist compared to 44.4% and 40.6% in early- and late-stage disease, respectively. Early-stage patients may seek care less often due to milder symptoms or limited awareness of progression while late-stage patients may be limited by mobility barriers or decreased access to specialty care through nursing facilities. Whether this affects patients outcomes remains unclear. Linking care patterns to outcomes across disease stages would clarify the clinical impact of these access gaps.

Medicaid patients, compared to commercially insured patients, had lower odds of having any neurologist care compared to no neurologist nor PCP care. Insurance type likely proxies for unmeasured factors, such as socioeconomic status, community-related factors, or health literacy, that shape access to neurological care. This disparity warrants further investigation given that many patients with HD depend on Medicaid or dual coverage to manage late-stage care.

This study has several limitations. First, we defined rurality at the county level. For some patients this may not accurately represent the rurality of their specific location within the county. Additionally, we were unable to assess differences in rural compared to frontier areas due to limitations in sample size. The Colorado APCD lacks key variables such as socioeconomic status, appointment wait times, and communication with providers outside clinic visits that may influence access to specialty neurological care. This study also utilized a cross-sectional design, therefore limiting understanding in how specialty care utilization may change over time. The method used to identify patients with HD in administrative claims data has not been formally validated. Therefore, patients with other conditions that mimic the symptoms of HD may have been included in the study population. Finally, characteristics used to determine disease stage may not have been present within the administrative claims data for some patients and use of this dataset to determine disease stage may mischaracterize a patient's true stage. Therefore, these results should be interpreted in the context of the limitations of claims-based disease-stage classification.

Future studies may utilize alternative datasets, such as Medicare claims, which have finer geographic detail and larger sample sizes that could better characterize barriers to neurological care and their effects on patient outcomes. A prospective study design could also be used to assess factors not included within claims data that influence access to specialized neurological care for patients with HD and how those factors influence patient health outcomes. Additionally, longitudinal data collection may increase understanding of how patterns of neurological care for patients with HD fluctuate over time and what factors may influence consistent or inconsistent access to neurological care. Due to the lack of disease modifying treatments and cures, patients with HD rely on their healthcare providers to help manage symptom progression. Until care patterns are linked to patient outcomes, the clinical consequences of these access gaps will remain unknown.

## Ethical compliance statement

This study was approved for exemption by the Colorado Multiple IRB Review Board (COMIRB #: 25-0475). Informed patient consent was not necessary for this work. We confirm that we have read the Journal's position on issues involved in ethical publication and affirm that this work is consistent with those guidelines.

## CRediT authorship contribution statement

**Amy C. Ogilvie:** Writing – original draft, Methodology, Funding acquisition, Formal analysis, Data curation, Conceptualization. **Emily Forbes:** Writing – review & editing, Methodology, Conceptualization. **Chun Chieh Lin:** Writing – review & editing, Methodology. **James F. Burke:** Writing – review & editing, Methodology. **Hillary D. Lum:** Writing – review & editing, Methodology, Conceptualization. **Sean M. Reed:** Writing – review & editing, Methodology, Conceptualization.

## Declaration of competing interest

The authors declare the following financial interests/personal relationships which may be considered as potential competing interests: Amy C Ogilvie reports financial support was provided by University of Colorado Denver Colorado Clinical and Translational Sciences Institute. Amy C Ogilvie reports financial support was provided by National Institute on Aging. Hillary D Lum reports financial support was provided by National Institute on Aging. Emily Forbes reports a relationship with Huntington's Disease Society of America that includes: funding grants. Emily Forbes reports a relationship with Movement Disorders Foundation that includes: funding grants. Emily Forbes reports a relationship with UniQure Inc. that includes: consulting or advisory. Chun Chieh Lin reports a relationship with American Academy of Neurology that includes: funding grants. Chun Chieh Lin reports a relationship with Genentech Inc. that includes: funding grants. Chun Chieh Lin reports a relationship with National Institutes of Health that includes: funding grants. James F Burke reports a relationship with Agency for Healthcare Research and Quality that includes: funding grants. James F Burke reports a relationship with National Institutes of Health that includes: funding grants. James F Burke reports a relationship with Genentech Inc. that includes: funding grants. Hillary D Lum reports a relationship with Alzheimer's Association that includes: funding grants. Hillary D Lum reports a relationship with National Institute on Aging that includes: funding grants. Hillary D Lum reports a relationship with Colorado Alzheimer's Disease and Related Dementia Action Coalition - Colorado Department of Public Health and Environment that includes: speaking and lecture fees. If there are other authors, they declare that they have no known competing financial interests or personal relationships that could have appeared to influence the work reported in this paper.
